# Hemophagocytosis induced by *Leishmania donovani* infection is beneficial to parasite survival within macrophages

**DOI:** 10.1371/journal.pntd.0007816

**Published:** 2019-11-18

**Authors:** Ayako Morimoto, Kazuyuki Uchida, James K. Chambers, Kai Sato, Jing Hong, Chizu Sanjoba, Yoshitsugu Matsumoto, Junya Yamagishi, Yasuyuki Goto

**Affiliations:** 1 Laboratory of Molecular Immunology, Department of Animal Resource Sciences, Graduate School of Agricultural and Life Sciences, The University of Tokyo, Bunkyo-ku, Tokyo, Japan; 2 Laboratory of Veterinary Pathology, Department of Veterinary Medical Sciences, Graduate School of Agricultural and Life Sciences, The University of Tokyo, Bunkyo-ku, Tokyo, Japan; 3 Research Center for Zoonosis Control, Hokkaido University, Sapporo, Japan; 4 Global Station for Zoonosis Control, GI-CoRE, Hokkaido University, Sapporo, Japan; University of Texas at El Paso, UNITED STATES

## Abstract

Visceral leishmaniasis (VL) is caused by parasitic protozoa of the genus *Leishmania* and is characterized by clinical manifestations such as fever, hepatosplenomegaly and anemia. Hemophagocytosis, the phenomenon of phagocytosis of blood cells by macrophages, is found in VL patients. In a previous study we established an experimental model of VL, reproducing anemia in mice for the first time, and identified hemophagocytosis by heavily infected macrophages in the spleen as a possible cause of anemia. However, the mechanism for parasite-induced hemophagocytosis or its role in parasite survival remained unclear. Here, we established an *in vitro* model of *Leishmania*-induced hemophagocytosis to explore the molecules involved in this process. In contrast to naïve RAW264.7 cells (mouse macrophage cell line) which did not uptake freshly isolated erythrocytes, RAW264.7 cells infected with *L*. *donovani* showed enhanced phagocytosis of erythrocytes. Additionally, for hemophagocytes found both *in vitro* and *in vivo*, the expression of signal regulatory protein α (SIRPα), one of the receptors responsible for the ‘don’t-eat-me’ signal was suppressed by post-transcriptional control. Furthermore, the overlapped phagocytosis of erythrocytes and *Leishmania* parasites within a given macrophage appeared to be beneficial to the parasites; the *in vitro* experiments showed a higher number of parasites within macrophages that had been induced to engulf erythrocytes. Together, these results suggest that *Leishmania* parasites may actively induce hemophagocytosis by manipulating the expression of SIRPα in macrophages/hemophagocytes, in order to secure their parasitism.

## Author summary

Parasites can manipulate host immune responses to build favorable environment to them. Because this parasite-driven immune modulation is often linked to symptoms in infected individuals, not only parasiticidal compounds but also immunological interventions limiting such the parasites’ abilities will serve as treatment options. In this study, we studied the mechanism and its role of hemophagocytosis (the phenomenon whereby macrophages engulf erythrocytes) caused by *Leishmania donovani*, a causative agent of VL. *In vitro* experiments revealed parasites have ability to directly disrupt macrophage’s recognition of self-cells, and that the induced engulfment of erythrocytes by *L*. *donovani* infection is beneficial to the parasites for their intracellular survival. These results suggest that *Leishmania* parasites actively induce hemophagocytosis by manipulating the ‘don’t-eat-me’ signal in macrophages for their survival. Although it is still to be determined how *Leishmania* parasites change the ‘don’t-eat-me’ signal in macrophages, our study may facilitate development of an immunotherapy which limits the change and lead to improvement of anemia due to hemophagocytosis as well as control of parasite survival.

## Introduction

Visceral leishmaniasis (VL), also known as kala-azar, is caused by parasitic protozoa of the genus *Leishmania*. Endemic countries of VL include India, Bangladesh, Nepal, Brazil, Ethiopia and Sudan. It is estimated that there are 50,000 to 90,000 new cases of VL and 26,000 to 65,000 deaths annually [1]. The parasites develop as promastigotes in the midgut of sand flies, the insect vector for *Leishmania*. Following a blood meal by sand flies, the parasites causing VL are transmitted to the mammalian host where they proliferate as amastigotes within macrophages in the spleen, liver and bone marrow. VL is characterized by clinical manifestations such as fever, weight loss, hepatosplenomegaly and anemia.

In a previous study, we established a mouse model of anemia during VL by *Leishmania (Leishmania) donovani*, and found that hemophagocytosis is up-regulated in the spleen of the infected mice [2]. Hemophagocytosis is the phenomenon whereby macrophages or histiocytes engulf erythrocytes and/or leukocytes in the bone marrow, liver or spleen [3]. Under normal conditions, macrophages phagocytose only senescent or injured blood cells. In some cases, however, engulfment of new and intact blood cells by hyper-activated macrophages or histiocytes can be observed at high frequency [3]. This up-regulated hemophagocytosis is observed during several infectious diseases including viral infections caused by Epstein-Barr (EB) virus and influenza virus [4,5], bacterial infections caused by *Salmonella* and *Mycobacterium* [6,7], and protozoan infections caused by *Babesia* and *Leishmania* [8,9].

Infection-associated hemophagocytosis may be induced through various mechanisms. IFN-γ and TNF-α play important roles in animal models of hemophagocytosis associated with infection by *Salmonella*, EB virus, lymphocytic choriomeningitic virus, cytomegalovirus and *Trypanosoma brucei* [10–14]. In fact, administration of IFN-γ alone can induce hemophagocytosis and anemia in mice [15]. On the other hand in *L*. *donovani* infection, hemophagocytosis is prominent in heavily infected macrophages, yet rarely found in the surrounding uninfected macrophages, suggesting that infection is directly responsible for making macrophages hemophagocytic, more so than activation through extracellular mediators like cytokines [2]. These results suggest that infection-associated hemophagocytosis is caused by a balance of extracellular and intracellular stimuli which varies with different infecting pathogens. For example, *T*. *brucei* shows extracellular parasitism in mammalian hosts while *Salmonella* is found in hemophagocytes similar to *L*. *donovani* [2,10,14].

Besides the pathological effect of induced hemophagocytosis, co-localization of intracellular pathogens and erythrocytes within a given macrophage may affect pathogen survival. The macrophage intracellular environment is low in pH, nutrient-poor and has higher levels of oxidative stress than the extracellular environment [16]. Phagocytosed RBCs may enhance nutrient availability within the macrophages and thus aid pathogen growth. In fact, it has been reported that RBC supplementation into macrophages increases the number of *Staphylococcus aureus* and *Salmonella typhimurium* bacteria within those cells [17]. However, the mechanism by which engulfed RBCs within *Leishmania*-infected macrophages are metabolized and the benefit of this to the parasites remain unknown.

Here, we have established an *in vitro* model of *L*. *donovani*-induced hemophagocytosis using a mouse macrophage cell line RAW264.7. Using this model, we explored the molecular mechanisms of hemophagocytosis as well as its benefit to the parasites. In addition, the molecules identified through the *in vitro* study were examined *in vivo* for their possible association with induced hemophagocytosis.

## Materials & methods

### Ethics statement

Animal experiments were reviewed and approved by an institutional animal committee at the Graduate School of Agricultural and Life Sciences, The University of Tokyo (Approval No. P14-930, P16-254 and P16-275). The experiments were performed in accordance with the Regulations for Animal Care and Use of the University of Tokyo, which were based on the Law for the Humane Treatment and Management of Animals, Standards Relating to the Care and Management of Laboratory Animals and Relief of Pain (the Ministry of the Environment), Fundamental Guidelines for Proper Conduct of Animal Experiment and Related Activities in Academic Research Institutions (the Ministry of Education, Culture, Sports, Science and Technology) and the Guidelines for Proper Conduct of Animal Experiments (the Science Council of Japan). Experiments using living modified organisms were reviewed and approved by an institutional animal committee at the Graduate School of Agricultural and Life Sciences, The University of Tokyo (Approval No. L19-015).

### Mice, cells and parasites

Male BALB/cA mice were purchased from Japan Clea, Tokyo, Japan. All mice were kept under specific pathogen-free conditions. The mice were used for experiments at the age of 6–8 weeks. Experimental infection of mice with *Leishmania (Leishmania) donovani* followed by hematological analyses and autopsy were performed as previously described [2].

Mouse macrophage cell line RAW264.7 cells, originally derived from tumors induced with Abelson leukemia virus, were obtained from ATCC (ATCC TIB-71). Cells were maintained at 37°C and 5% CO_2_ in Dulbecco’s Modified Eagle Medium (DMEM: Sigma-Aldrich, Japan) containing 10% heat-inactivated fetal bovine serum (HI-FBS) (Thermo Fisher Scientific, USA), 100 U/ml penicillin and 100 μg/ml streptomycin (Thermo). The supplemented DMEM, called complete DMEM, was used for macrophage culture unless otherwise noted.

In order to harvest mouse peritoneal exudate cells (PECs), 1 ml of 10% thioglycolate medium (Nissui Pharmaceutical, Japan) was injected intraperitoneally into BALB/c mice. At 72 h post-inoculation the mice were sacrificed, and PECs were harvested from their peritoneal cavity by using 5 ml of PBS. The PECs were washed three times with PBS before being used for the following experiments. Bone marrow-derived macrophages (BMDMs) were generated by cultivating bone marrow cells of BALB/c mice in complete DMEM supplemented with 25 ng/ml recombinant M-CSF (PeproTech, USA) for 6 days at 37°C and 5% CO_2_ in a 12-well plate. The medium was changed once with fresh one on Day 4.

Promastigotes of *L*. *donovani* (MHOM/NP/03/D10; gifted from National BioResource Project at Nagasaki University [18]) were cultured in medium TC199 (Nissui) supplemented with 10% HI-FBS and 25 mM HEPES buffer (MP Biomedicals, USA) at 25°C. For some experiments, promastigotes were stained with CFSE (Dojindo Molecular Technologies, Japan). *L*. *donovani* promastigotes (2 × 10^6^ cells) were incubated at room temperature for 30 min in 200 μl of RPMI1640 medium (Sigma) containing 50 μg/ml of CFSE. The stained promastigotes were washed three times with RPMI1640 medium and used for *in vitro* infection experiments.

*L*. *donovani* expressing EGFP (Ld/*egfp*) was generated by electroporation of the p6.5-EGFP vector into *L*. *donovani* promastigotes as previously reported [19]. Ld/*egfp* promastigotes were maintained at 25°C in medium TC199 containing 10% HI-FBS, 25 mM HEPES and 20 μg/ml tunicamycin (FUJIFILM Wako Pure Chemical, Japan).

### *In vitro* erythrophagocytosis assay

As an initial experiment, establishment of *L*. *donovani*-induced hemophagocytosis was performed using *in vitro* cultured macrophages. RAW264.7 cells were seeded at 4 × 10^4^ cells per well in 16 well chamber slide glasses (Thermo). After 2 h incubation at 37°C, 2 × 10^6^ of Ld/*egfp* promastigotes were added to each well followed by incubation for 6 h at 37°C. Next, wells were washed with DMEM three times and cultured with 20 U/ml IFN-γ (eBioscience, USA) for 16 h at 37°C. Heparinized blood from naïve mice was washed with DMEM twice by centrifugation at 200 ×*g* for 10 min. Next, 2 μl of blood was labeled with 200 μl of CytoRed (Dojindo) in 400 μl of DMEM for 1 h at 37°C. After centrifugation and washing twice, 8 × 10^5^ of RBCs were added to each well and incubated for 2 h at 37°C_._ Wells were then washed with PBS three times and fixed with 4% paraformaldehyde (FUJIFILM Wako) for 20 min. After washing with PBS, cells were stained with Hoechst33342 (Dojindo) for 10 min. Glasses were washed with PBS and mounted with cover slips and finally, fluorescence observation was performed.

In the second experiment, we investigated whether or not *L*. *donovani*-induced up-regulated hemophagocytosis specifically affects RBCs. RAW264.7 (1.2 × 10^5^ cells) were incubated with 6 × 10^6^ of live *L*. *donovani*, frozen/killed *L*. *donovani* or polystyrene beads for 6 h. Afterwards the wells were washed with DMEM three times and the macrophages were added with fresh complete DMEM. The wells were then incubated for an additional 66 h. Then, either CytoRed-stained RBCs or FITC-conjugated polystyrene beads (Polysciences Inc., USA) were added. After a 6 h incubation, the cells were washed, fixed and observed as described above, and the ratios of phagocytes engulfing RBC or polystyrene beads were calculated respectively. For quantitative analysis, macrophages harboring erythrocytes or polystyrene beads were counted in 5 random microscopic fields at 200× magnification.

In order to investigate whether *L*. *donovani* induces hemophagocytosis of RBCs in primary macrophages, PECs (1 × 10^5^ cells) were incubated with 5 × 10^6^ of CFSE-labeled *L*. *donovani* for 24 h in RPMI 1640 medium supplemented with 10% HI-FBS, 100 U/ml penicillin and 100 μg/ml streptomycin (complete RPMI 1640 medium) at 37°C. Afterwards the wells were washed with complete RPMI 1640 medium three times and the macrophages were added with fresh complete RPMI 1640 medium. Then, 2 × 10^6^ CytoRed-stained RBCs were added. After a 2 h incubation, the cells were washed and observed as described above, and a proportion of phagocytes engulfing RBC were calculated.

BMDMs were also used for experiments of *L*. *donovani*-induced phagocytosis. BMDMs were incubated with CFSE-labeled *L*. *donovani* (1:20 ratio) for 24 h. Afterwards the wells were washed with DMEM three times and the macrophages were added with fresh complete DMEM. Then, CytoRed-stained RBCs were added (macrophage:RBC = 1:20). After a 2 h incubation, the cells were washed and observed as described above, and a proportion of phagocytes engulfing RBC were calculated.

### Transcriptome analysis

Three hundred thousand cells of RAW264.7 were cultured in complete RPMI 1640 medium at 37°C. Cells were infected with 1.5 × 10^7^ cells of *L*. *donovani* and incubated for 72 h at 37°C. Total RNA was extracted using TRIzol reagent (Invitrogen Inc., USA) according to the manufacture’s instruction. RNA-seq and subsequent transcriptome analysis was performed in order to identify the genes associated with macrophage hemophagocytic activity, and changes in mRNA expression levels after *L*. *donovani* infection.

### Western blotting

Three hundred thousand cells of RAW264.7 were cultured in complete RPMI 1640 medium at 37°C. The cells were infected with 1.5 × 10^7^ cells of *L*. *donovani* and incubated for 24h at 37°C. After washing three times with PBS, the macrophages were lysed in RIPA Buffer (50 mM Tris-HCl, 150 mM NaCl, 1% NP-40, 0.5% sodium deoxycholate, 0.1% SDS) supplemented with protease inhibitor cocktail (Sigma Aldrich). Cell lysates were diluted with SDS sample buffer, boiled for 5 min and separated by electrophoresis on an SDS-containing 4–20% Tris-HCl gradient gel (Thermo), then transferred to a polyvinylidene difluoride membrane (GE Healthcare Bio-Sciences, USA). After blocking with Block Ace (Sumitomo Dainippon Pharma, Japan), the membrane was probed with anti-SIRPα antibody (RayBiotech, USA; 1:2,000 dilution) and anti-GAPDH antibody (GeneTex, USA; 1:2,500 dilution) diluted with PBS containing 0.05% Tween 20 (PBS-T) plus 10% Block Ace. After washing the membrane with PBS-T three times, it was probed with horseradish peroxidase (HRP)-linked donkey anti-rabbit IgG antibody (GE Healthcare) at 1:5,000 dilution with PBS-T containing 10% Block Ace. Bands were visualized by an enhanced chemiluminescence detection system (GE Healthcare) and analyzed by LAS-3000 mini (Fujifilm, Japan). Densitometric analysis was performed using Image J software from the National Institute of Health.

### Histological and Immunohistochemical analyses

The tissues collected at the time of sacrifice were fixed with buffered 20% formalin (Sumitani Shoten Co., Ltd, Japan) and embedded in paraffin. Histological and immunohistochemical staining of tissue sections using rat anti-mouse F4/80 (AbD Serotec, UK) or rabbit anti-SIRPα (Abcam, UK) was performed as previously described [2].

### Assays of *Leishmania* survivability in macrophages

To examine whether the existence of RBC in macrophages affects *Leishmania* amastigotes survivability, 2 × 10^4^ cells of RAW264.7 were added with either glutaraldehyde-treated 1 × 10^7^ cells of RBC or polystyrene beads at 37°C, and were then infected with 1 × 10^6^ cells of *L*. *donovani* Medium was changed with fresh mediums every day. After incubation for 72 h, cells were washed, fixed with methanol for 5 min and stained with 5% Giemsa solution for 25 min. The ratio of macrophages infected with *Leishmania* amastigotes to the total number of macrophages, and the average number of phagocytosed amastigotes in infected macrophages were calculated through microscopic observation of at least 500 stained cells at 1,000× magnification.

### RT-PCR

For the quantitative RT-PCR, RNA and DNA was extracted following the manufacturers’ instructions using AllPrep DNA/RNA Mini Kit (QIAGEN, Netherlands). The concentration of total RNA and DNA was measured by DU730 Life Science UV/vis spectrophotometer (Beckman Coulter, USA), and 300 ng of total RNA was used as the template for the synthesis of 20 ul of cDNA. A tube containing 1.25 μM oligo (dT)_16_, and 0.5 mM dNTPs (Thermo) with template RNA was incubated for 5 min at 65°C. After adding 5x first strand buffer and 10 mM DTT (Thermo), 200 U M-MLV (Thermo) was added and the tube was incubated for 50 min at 37°C followed by a further 15 min at 70°C. cDNA was synthesized and analyzed for expression of heme oxygenase 1 (*Hmox1)*, ferroportin *(Fpn)*, ferritin *(Fth)* and DNA for *Leishmania gapdh*. The designed primers are listed in S1 Table. Real-time polymerase chain reaction (PCR) assay was carried out using 30 ng of cDNA or 80 ng of DNA as the template and 10 μl of SYBR Select Master Mix (Thermo) on the ABI Prism 7000 Sequence Detection System (Thermo). Data was analyzed by 2^-ΔΔCt^ methods and normalized by murine *Gapdh*. The thermal cycling conditions for the PCR were 94°C for 10 min, followed by 40 cycles at 94°C for 15 sec and 60°C for 1 min.

### Statistical analyses

Statistical comparisons were performed by one-way ANOVA followed by Bonferroni’s multiple comparison test or unpaired *t* test with GraphPad Prism 6 software (GraphPad Software, Inc., La Jolla, USA). A difference between groups was considered as statistically significant when the *P* value was less than 0.05.

## Results

### *L*. *donovani* infection induces hemophagocytosis of erythrocytes by macrophage

**Fig 1:**
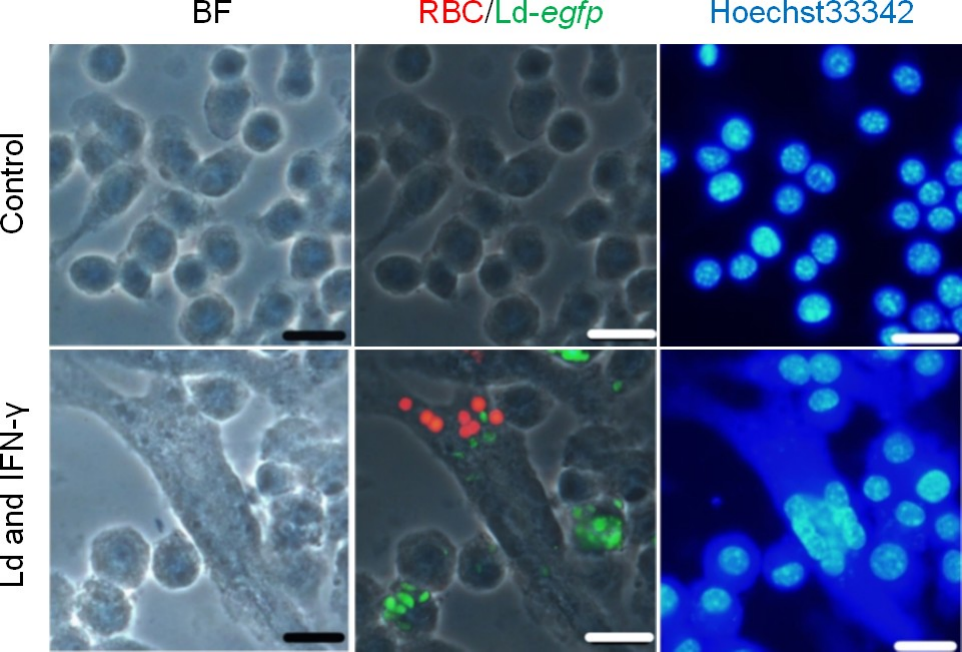
Up-regulated uptake of erythrocytes by macrophages infected with *L*. *donovani in vitro*. A representative fluorescence microscopy image of RAW264.7 cells cultured with medium only (upper panel) or with *L*. *donovani* and IFN-γ (lower panel) are shown; Ld*/egfp*, green; RBCs, red; nuclei, blue. This experiment was conducted once. Scale bar: 20 μm.

**Fig 2:**
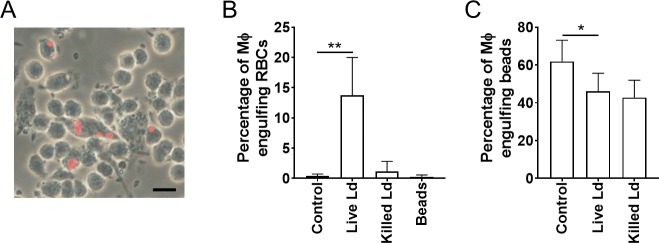
Up-regulated uptake of erythrocytes but not polystyrene beads by RAW264.7 cells infected with *L*. *donovani*. RAW264.7 cells were cultured with live/killed *L*. *donovani* followed by incubation with RBCs or polystyrene beads. (A) A representative image of *L*. *donovani*-infected RAW264.7 cells phagocytosing RBCs (red) is shown. (B, C) The percentage of phagocytes engulfing RBCs (B) or polystyrene beads (C) were counted. The mean + SD of at least five fields are shown. These are representatives of three independent experiments with similar results. *P < 0.05, **P < 0.01 by one-way ANOVA followed by Bonferroni's multiple comparisons test.

The hemophagocytic activity of RAW264.7 cells was tested to determine whether *L*. *donovani* infection actually induces hemophagocytosis by macrophages *in vitro*. In RAW264.7 cells incubated with medium only, hemophagocytosis was rarely observed (Fig 1, upper panels). In contrast, when RAW264.7 cells were cultured with Ld/*egfp* and IFN-γ, it was observed that more cells had phagocytosed erythrocytes (Fig 1, lower panels). It is noted that hemophagocytosis was prominent in cells harboring parasites and that these cells often had a multinuclear characteristic. In order to determine if active infection with *L*. *donovani* is indispensable for the up-regulated hemophagocytosis, RAW264.7 cells were treated with live parasites, killed parasites or polystyrene beads (as a representative control of non-*Leishmania* particles), before being examined for hemophagocytic activity. As shown in Fig 2B, the percentage of macrophages engulfing RBCs was higher in *L*. *donovani*-infected cells (13.7 ± 6.3%) than that of either uninfected cells, cells treated with killed *L*. *donovani*, or cells treated with beads (0.4 ± 0.3%, 1.1 ± 1.7%, 0.3 ± 0.3%, respectively).

Next, in order to examine whether *L*. *donovani* induces hemophagocytosis or up-regulates general phagocytic activity, infected macrophages were examined for phagocytosis of polystyrene beads as a representative control of non-RBC particles. In contrast to the results of the RBC engulfment experiment, the proportion of macrophages engulfing polystyrene beads was not up-regulated in cells infected with either live or killed *L*. *donovani* compared with uninfected cells (46.0 ± 9.6%, 42.8 ± 9.1%, 61.9 ± 11.2%, respectively; Fig 2C).

**Fig 3:**
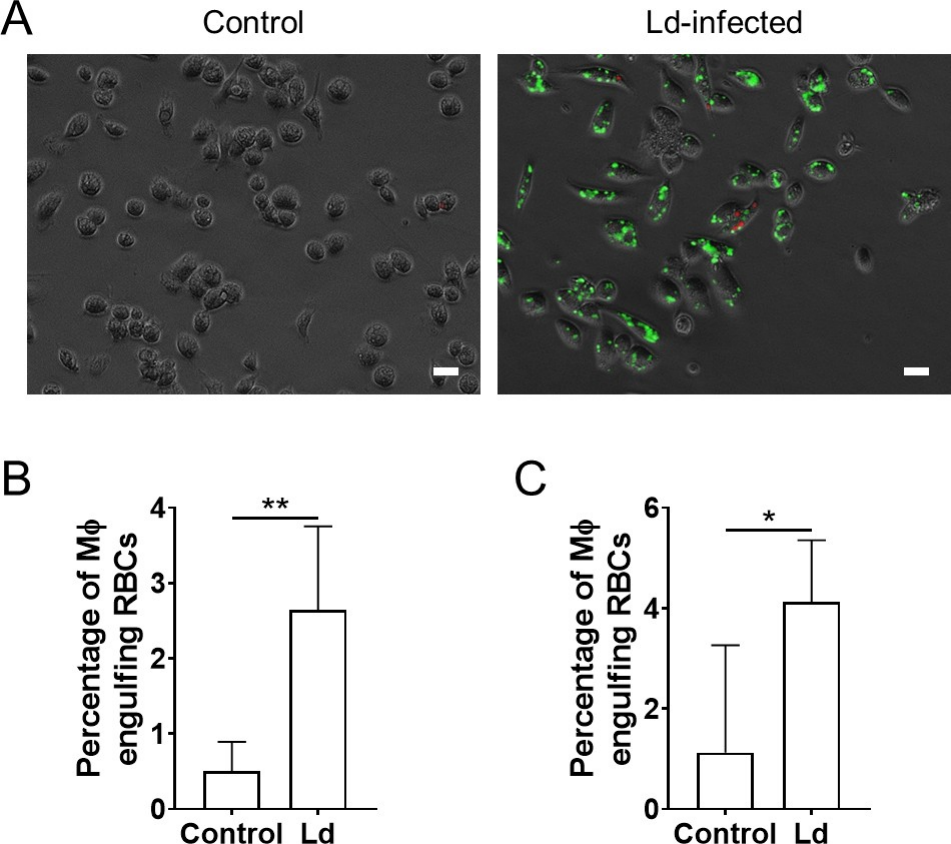
Up-regulated uptake of erythrocytes by primary macrophages infected with *L*. *donovani*. Peritoneal exudate cells (PECs) or bone marrow-derived macrophages (BMDMs) were cultured with *L*. *donovani* followed by incubation with RBCs. (A) Representative images of uninfected PECs and *L*. *donovani* (green)-infected PECs followed by incubation with RBCs (red) are shown. (B, C) The percentage of phagocytes engulfing RBCs were counted for PECs (B) and BMDMs (C), respectively. The mean + SD of at least four fields are shown. These are representatives of two independent experiments with similar results. *P < 0.05, **P < 0.01 by unpaired *t* test.

In order to examine whether *L*. *donovani* can induce hemophagocytosis not only in RAW264.7 cells but also in primary macrophages, PECs and BMDMs infected with the parasites were examined for phagocytosis of RBCs. Similar to the results of RAW264.7 cells, uninfected PECs and BMDMs showed minimal phagocytosis of RBCs, while those cells infected with *L*. *donovani* parasites showed elevated phagocytosis of RBCs (Fig 3).

### *L*. *donovani* infection suppresses the expression of SIRPα in macrophages

To understand what changes may be inducing macrophage hemophagocytosis, RNA-seq analysis of naïve or *L*. *donovani-*infected RAW264.7 cells was carried out. Familial hemophagocytic lymphohistiocytosis (f-HLH) is a heterogeneous autosomal recessive disorder representing up-regulated hemophagocytosis which are associated with specific genes [3]. First, the mRNAs levels of nine genes associated with f-HLH [3] were examined. None of the nine genes showed significantly different levels of expression (adjusted *P* < 0.05) between the uninfected and infected RAW264.7 cells (S2 Table). Next, the mRNA levels of 38 genes involved in non-specific phagocytosis and 16 genes associated with inhibition of phagocytosis [20,21] were examined. There were several genes whose levels of mRNA expression were significantly (adjusted *P*< 0.05) different post-infection, including *Fcgr1* and *Cd36* which were up-regulated and *Cd33* which was down-regulated. However, there was no consistent trend seen within the promoting or inhibitory gene groups, and none of the differently expressed genes were closely linked to hemophagocytosis in previous studies (S3 Table and S4 Table).

**Fig 4:**
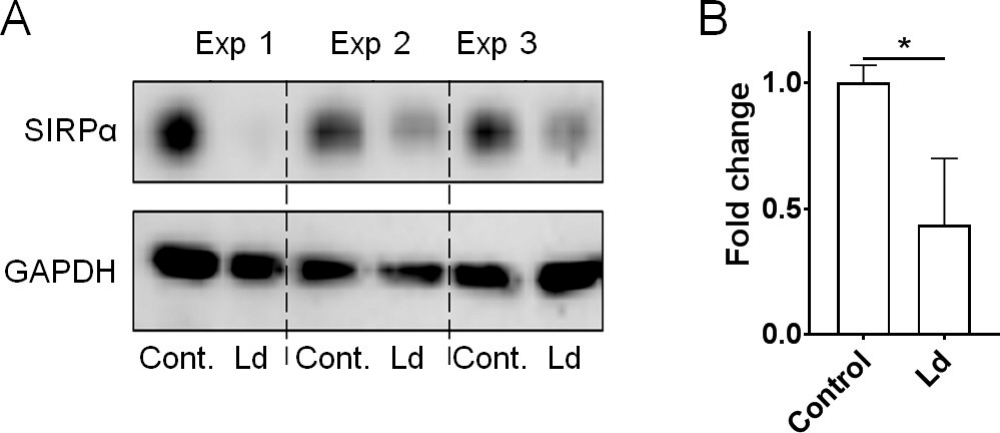
Down-regulation of SIRPα in *L*. *donovani*-infected macrophages. (A) Immunoblotting for SIRPα in naïve/*L*. *donovani*-infected RAW264.7 cells. The results of three independent experiments are shown. (B) Densitometric analysis with Image J software also showed decreased SIRPα in the infected macrophages. The mean and SD for each group are shown. *P < 0.05 by unpaired *t* test.

Since transcriptome analysis did not demonstrate candidate molecules responsible for *L*. *donovani*-induced up-regulation of hemophagocytosis, we decided to focused on signal regulatory protein α (SIRPα) which is the only receptor which is known to be involved in inhibiting RBC uptake through ligation to CD47 [22–24]. Western blot analysis showed that SIRPα expression is suppressed in *L*. *donovani*-infected RAW264.7 cells when compared with that of naïve RAW264.7 cells (Fig 4). In contrast, mRNA expression levels for *Sirpa* were comparable between *L*. *donovani*-infected and naïve RAW264.7 cells as shown in S4 Table.

**Fig 5:**
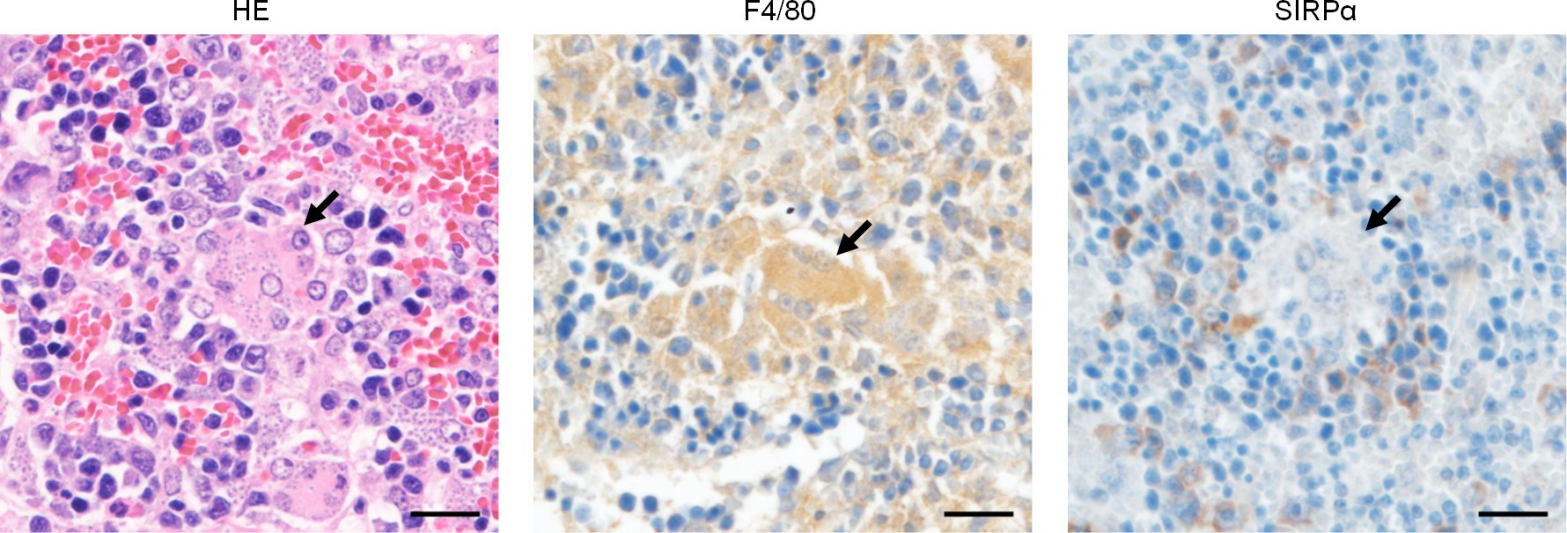
Low SIRPα expression on MGCs in the spleen of *L*. *donovani*-infected mice. Sections from the spleen of *L*. *donovani*-infected mice at 24 weeks post-infection were stained with Hematoxylin/Eosin, or immunohistochemically stained with anti-F4/80 or anti-SIRPα antibody, followed by counterstaining with hematoxylin. Arrows point to parasitized MGCs. Scale bars, 20 μm.

To examine whether SIRPα expression is also down-regulated in hemophagocytes found in the spleen of *L*. *donovani*-infected mice, histological and immunohistochemical staining was performed on the spleen from either naïve mice or 24 week-infected mice by using anti-F4/80 antibody or anti-SIRPα antibody. When focusing on multinucleated giant cells (MGCs) in the spleen of *L*. *donovani*-infected mice, F4/80 staining of MGCs harboring amastigotes was positive and its signal intensity was similar to that of the surrounding uninfected cells (Fig 5). In contrast, the SIRPα signal was weaker in infected MGCs than in the surrounding non-MGCs (Fig 5). In naïve mice, no MGCs were observed in the red pulps of the spleen where the positive signals of F4/80 [2] and SIRPα (S1 Fig) were detected.

### RBC engulfment is beneficial to *L*. *donovani* survival in macrophages

**Fig 6:**
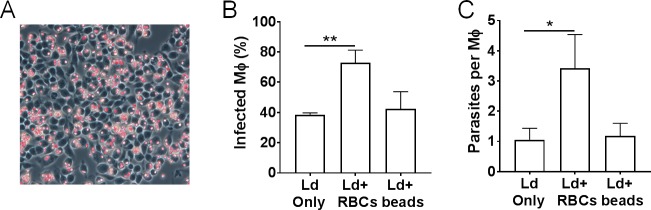
Increased survival of *L*. *donovani* in RBC-supplemented macrophages. (A) A representative image of uninfected macrophages (Mφ) engulfing glutaraldehyde-treated RBCs, showing highly efficient uptake of the treated RBCs. (B and C) Glutaraldehyde-treated RBCs or polystyrene beads were added to RAW264.7 cells infected with *L*. *donovani*. After 66 h, the number of infected Mφ (B) and that of amastigotes per Mφ (C) cultured in medium-only (LD only), RBC-supplemented medium (LD + RBCs) or bead-supplemented medium (LD + beads) were counted on Giemsa-stained samples. The mean + SD of triplicate are shown. These are representative of four independent experiments with similar results. *P < 0.05, **P < 0.01 by one-way ANOVA followed by Bonferroni's multiple comparisons test.

The influence of RBC engulfment on parasite survival was examined using the *in vitro* system. In order to explore this in a time-efficient manner, RBC engulfment by macrophages was artificially induced using glutaraldehyde (GA)-fixed RBCs in the study rather than naïve RBCs as in the previous experiments. In a preliminary experiment where uninfected RAW264.7 cells were incubated with GA-RBCs at 1:10 ratio for 1 h, a high frequency of macrophages engulfing RBCs was observed (Fig 6A).

Macrophages were first infected with *L*. *donovani* and then incubated with either medium alone, medium containing GA-RBCs or medium containing polystyrene beads (as a non-RBC control). At 72 h, the proportion of infected macrophages as well as the average number of amastigotes within a given infected macrophage were calculated. At 72h post-infection, approximately 80% of GA-RBC-treated macrophages had ingested RBCs and around 70% had ingested beads. The proportion of infected macrophages in the medium-only control group was 38.55 ± 1.16% (Fig 6B). In contrast, this was higher in the GA-RBC-treated group in which 72.91 ± 8.25% of macrophages were infected. The bead-treated macrophages showed similar infection rates to the control group (42.34 ± 11.34%). In addition, the number of amastigotes per macrophage in RAW264.7 cells exposed to GA-RBCs was higher (3.43 ± 1.11) than those in macrophages exposed to beads or in untreated macrophages (1.06 ± 0.38 and 1.18 ± 0.42, respectively) (Fig 6C).

### Up-regulated degradation of heme in macrophages infected by *L*. *donovani* parasites

**Fig 7:**
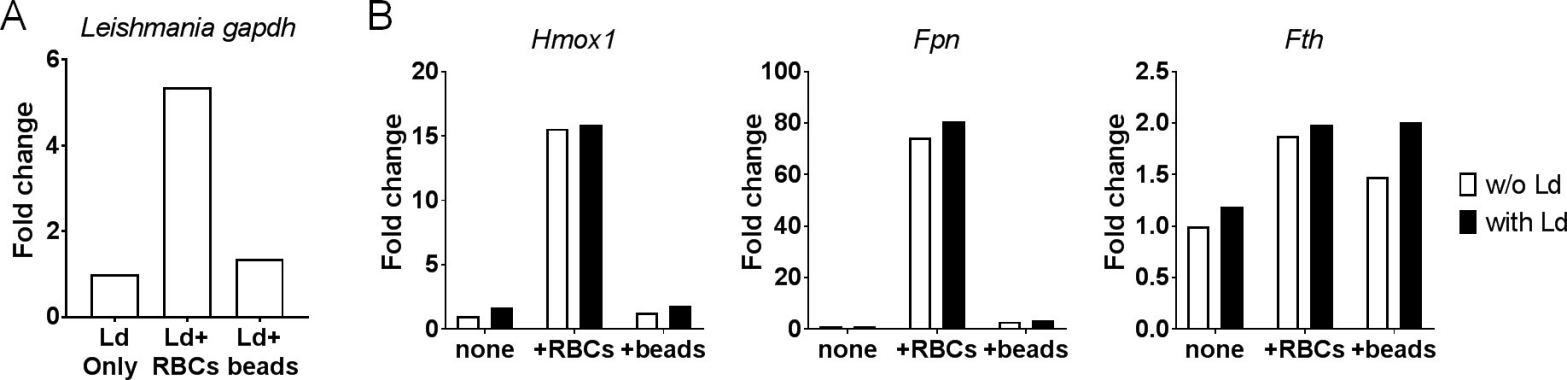
Increased *Hmox1* gene expression in RBC-supplemented RAW264.7 cells. *L*. *donovani*-infected RAW264.7 cells were cultured in medium-only (LD only), RBC-supplemented medium (LD + RBCs) or bead-supplemented medium (LD + beads). (A) *Leishmania gapdh* DNA were quantified by qPCR. (B) Murine *Hmox1*, *Fpn* and *Fth* mRNA were quantified by qPCR. The values show fold change in expression levels after normalization with murine *Gapdh* levels. The data are representative of two independent experiments with similar results.

The levels of parasite DNA as well as mRNA corresponding to genes involved in heme/iron metabolism were examined *in vitro* in macrophages exposed to GA-RBCs/polystyrene beads/medium-only. Higher levels of parasite DNA were detected in GA-RBC-treated macrophages than the others (Fig 7A), reproducing the findings of the previous section. In the parasite-friendly condition, mRNA expression of *Hmox1* and *Fpn*, iron exporter gene, was up-regulated when compared to the control group macrophages. Meanwhile, the expression of *Fth*, a gene responsible for iron storage, was unaffected (Fig 7B). The up-regulation of *Hmox1* and *Fpn* gene expression appeared to be more dependent on RBC uptake than *Leishmania* infection as this up-regulation was also found in uninfected macrophages that had been treated with GA-RBCs (Fig 7B).

## Discussion

As described in the previous report, *L*. *donovani* infection and the subsequent hyper-activation of macrophages is suspected to be an important factor for causing macrophages to acquire hemophagocytic activity *in vivo* [2]. In line with this, *Leishmania*-induced hemophagocytosis and subsequent anemia can be reversed in infected mice using chemotherapy with liposomal amphotericin B (S2 Fig). In order to completely eliminate any influence from autoantibodies or any damage to RBC on the hemophagocytosis observed *in vivo*, an *in vitro* model for hemophagocytosis was established using the macrophage cell line RAW264.7, *L*. *donovani* parasites and RBCs from naïve mice. Results from the *in vitro* experiments demonstrated that *L*. *donovani* has the ability to induce macrophages to phagocytose healthy RBC.

In the first experiment, IFN-γ was used as an additional inducer for of macrophage hemophagocytosis *in vitro*. This was done because our *in vivo* experiments showed that hemophagocytosis does not occur in nude mice (S3 Fig), suggesting the importance of T cell signaling for hemophagocytosis to occur. However, we eventually found that additional stimulation with cytokines is dispensable for *L*. *donovani*-induced hemophagocytosis (Figs 2 and 3). One of the possible reasons for this discrepancy may be that there are differences in characters between RAW264.7 cells and splenic macrophages found in the infected mice. For example, macrophages in the spleen appear to be heterogenous. In our previous study, the hemophagocytes were characterized as a MOMA2-positive population which was not the major cell type amongst the splenic macrophages [2]. Although the target molecule for the MOMA-2 antibody has not been characterized yet, it is possible that the T-cells may activate a subpopulation of splenic macrophages and thus induce the MOMA2-positive status, whereas RAW264.7 cells are already in this active state, even without stimulation from T-cells. Furthermore, although both liver and splenic macrophages were found to be parasitized in *L*. *donovani*-infected mice, hemophagocytes and MGCs were not found in the liver [2], suggesting that parasitization is not the sole factor making contributing to the transformation of macrophages into hemophagocytes. Nonetheless, our *in vitro* study clearly demonstrated that *Leishmania* infection can induce hemophagocytosis by macrophages: *L*. *donovani*-infected RAW264.7 cells engulfed RBCs from naïve mice, whilst uninfected RAW264.7 cells did not. This supports our hypothesis that autoantibodies and damage to RBCs are not the major factors responsible for *L*. *donovani*-induced hemophagocytosis.

Another possible reason for the discrepancy may be the total stimulus intensity. Although IFN-γ alone can cause hemophagocytosis in mice, the amount of IFN-γ needed is quite high; more than 2,500 pg/ml in serum [15]. In contrast, serum IFN-γ of 24-week-infected BALB/cA mice was only around 100 pg/ml (S4 Fig). This implies that IFN-γ is not the sole factor causing hemophagocytosis in *L*. *donovani* infection, yet it is still possible that this cytokine, together with intracellular signals from *Leishmania* parasites triggers the transformation of splenic macrophages into hemophagocytes.

The successful establishment of an *in vitro* model for *L*. *donovani*-induced hemophagocytosis has simplified the analyses of mechanisms involved in this phenomenon. Naïve and *L*. *donovani*-infected RAW264.7 cells may simply be compared in order to identify molecules involved in hemophagocytosis. Thus, transcriptome analysis was initially performed to identify any alterations in mRNA levels in macrophages after infection. However, no significant changes were observed in the expression of genes associated with f-HLH (S2 Table). Additionally, the analysis of genes encoding receptors involved with induced phagocytosis did not provide clear conclusions regarding *L*. *donovani*-induced hemophagocytosis. Although some genes including *Fcgr1* and *Cd36* were significantly up-regulated in the infected macrophages, this is unlikely to explain the up-regulated hemophagocytosis caused by *L*. *donovani*, because the influence of autoantibodies or damage to RBCs had already been accounted for. Overall, the transcriptome analysis was not successful for identification of molecules responsible for *L*. *donovani*-induced hemophagocytosis. However, it indicated that this phenomenon is dependent on molecules that are not regulated at the transcriptional level, but instead for example, by proteolysis.

The aforementioned was one of the reasons we chose to focus on SIRPα next. Another reason was because that the enhanced uptake of erythrocytes by the infected cells is not due to up-regulation of non-specific phagocytosis but rather is dependent on erythrocyte-biased mechanisms (Fig 2). SIRPα is one of the phagocytosis-inhibitory receptors and is the only one known to be involved with recognition of RBCs, through the ligand molecule CD47 [22–24]. It has been reported that SIRPα-KO mice show reduced levels of RBCs, hematocrit and hemoglobin in peripheral blood due to erythrophagocytosis by splenic macrophages [24]. Additionally, SIRPα expression in macrophages or monocytes can be regulated by metalloproteinase cleavage upon stimulation [25,26]. In this study, we demonstrated the down-regulation of SIRPα in *L*. *donovani*-infected RAW264.7 cells *in vitro*, as well as in MGCs in the spleen of infected mice *in vivo*. These results suggest that the down-regulation of SIRPα in macrophages induced by *L*. *donovani* infection disrupts the receptor-ligand interactions with CD47 found on RBCs. Thus, the appropriate ‘don’t-eat-me’ signal is lost, resulting in hemophagocytosis. Although CD47-SIRPα signaling is currently the only known mechanism for limiting hemophagocytosis, there may be other molecules involved in this self-recognition. It is necessary to explore if SIRPα down-regulation is actually involved in enhanced hemophagocytosis during experimental VL in the future by generating genetically modified mice harboring cleavage-resistant SIRPα mutant. This will also answer if hemophagocytosis is the major cause of anemia in the experimental model.

So, does the ability of *L*. *donovani* to make infected macrophages hemophagocytic benefit the parasites? Indeed, by using an *in vitro* model of induced hemophagocytosis, we found that engulfment of RBCs by parasitized macrophages is advantageous to *Leishmania* parasites (Fig 6). This coincides with the finding that hemophagocytosis occurs frequently by heavily infected macrophages *in vivo* [2]. Although we have not shown direct evidence as to how hemophagocytosis benefits *Leishmania*, there are some reports indicating that products from RBC degradation can support the parasites’ survival [27].

One possible explanation may be that *Leishmania* parasites derive nutrients including heme and heme-contained iron from RBCs. Heme and iron are essential for many cellular processes including DNA replication, oxygen transport and the mitochondrial electron transport chain, drug and steroid metabolism, and transcription and regulation of antioxidant defense enzymes [28]. However, trypanosomatid parasites lack a complete heme synthesis pathway [29] and phagocytosed RBCs within the infected macrophages may be a good source for heme acquisition by intracellular *Leishmania* amastigotes. Iron is also indispensable for the efficient survival of both intracellular and extracellular forms of *Leishmania* species [30,31]. Although the acquisition and utilization of heme/iron by *Leishmania* are not well understood, recent works have identified three *L*. *amazonensis* proteins and one *L*. *donovani* protein, associated with iron/heme transport, and their elevated levels of expression in macrophage phagolysosomes [32–35]. In addition, Paramchuk *et al*. described that *Leishmania chagasi* expresses Fe-superoxide dismutase (SOD) and parasites lacking this gene could not be obtained [36,37]. Similar findings have been obtained with *L*. *tropica* [38]. *L*. *donovani*, *L*. *aethiopica* and *L*. *major* also have Fe-SOD genes [39,40].

RBC engulfment by macrophages may also benefit the parasites by weakening the immune status of host. Hand *et al*. described that macrophages supplemented with erythrocytes produce lower intracellular levels of superoxide, resulting in the successful proliferation of bacteria [17]. After RBC degradation, heme is broken down by HMOX1 into iron, biliverdin and carbon monoxide. Some reports have investigated the relationship of HMOX1 and *Leishmania* [41–45], describing that higher levels of HMOX1 in macrophages during *Leishmania* infection correlate with an increase in parasite load. Increased HMOX1 also reduces oxidative stress in cells via degradation of NADPH [46]. Carbon monoxide has not been directly linked to *Leishmania* survival, but it is known to suppress inflammation during *Plasmodium* infection in mice [47]. Our finding that mRNA levels of *Hmox1* are higher in RBC-supplemented macrophages than non-RBC-supplemented macrophages (Fig 7), implies that the degradation of RBCs/hemoglobin creates *Leishmania*-friendly environment within macrophages.

## Supporting information

S1 Fig A representative image of the spleen of naïve BALB/c mice immunohistochemically stained with anti-SIRPα antibody. Bar, 200 μm.
(TIF)

S2 Fig Improved anemia and reduced hemophagocytosis by antileishmanial drug BALB/cA mice were infected with 1 × 107 *L*. *donovani* promastigotes. At 24 weeks post-infection, the mice were administered with 200 μg of liposomal amphotericin B (AmBisome; Dainippon Sumitomo Pharma, Japan) for 5 days. The mice were sacrificed 10 days after completing treatment to examine organ weights and parasite burden of the spleen (A and B) and the liver (C and D), in addition to hematocrit (E), hemoglobin (F), peripheral blood red blood cell counts (G), proportion of polychromatic erythrocytes in peripheral blood (H) and percentage of hemophagocytes out of total number of splenic macrophages (I). White, black and grey bars represent naïve, infected/untreated and infected/AmBisome-treated mice respectively. The mean and SD of 5 mice in each group are shown. This experiment was conducted once. *P < 0.05, **P < 0.01 by one-way ANOVA followed by Bonferroni's multiple comparisons test (for A, C, E to I) or unpaired t test (for B and D); ns, not significant.
(TIF)

S3 Fig No anemia in *L*. *donovani*-infected nude mice. BALB/c mice and BALB/c-nu/nu (nude) mice (Clea) were infected with 1 × 107 *L*. *donovani* promastigotes by intravenous injection into the tail vein. At 24 weeks post-infection, the infected mice as well as age-matched naïve mice were sacrificed to examine hematocrit (A), hemoglobin (B) and peripheral blood cell counts (C). The mean and SD of at least 4 mice in each group are shown. (D) A representative image of a HE-stained section of the spleen harvested from L. donovani-infected nude mice is shown. These are representative of two independent experiments with similar results. **P < 0.01 by two-way ANOVA followed by Bonferroni's multiple comparisons test; ns, not significant.
(TIF)

S4 Fig BALB/c mice were infected with 1 × 107 *L*. *donovani* promastigotes by intravenous injection into the tail vein. At 24 weeks post-infection, serum samples of naïve and infected mice were collected, and serum levels of IFN-γ were determined by using Mouse IFN gamma ELISA Ready-SET-Go! Kit (eBioscience, detection limit = 15 pg/ml). The mean and SD of 5 mice in each group are shown. ND, not detected. This experiment was conducted once.
(TIF)

S1 Table Primers used in this study. (DOCX)

S2 Table mRNA levels of fHLH-involved genes in *L*. *donovani*-infected macrophages. (DOCX)

S3 Table mRNA levels of phagocytosis-promoting receptor genes in *L*. *donovani*-infected macrophages. (DOCX)

S4 Table mRNA levels of phagocytosis-inhibitory receptor genes in *L*. *donovani*-infected macrophages. (DOCX)
