# Supplementary figures and images for: Hemophagocytosis induced by Leishmania donovani infection is beneficial to parasite survival within macrophages
Source: PLoS Negl Trop Dis. 2019 Nov 18;13(11):e0007816. doi: 10.1371/journal.pntd.0007816 (PMC6886864; doi:10.1371/journal.pntd.0007816)

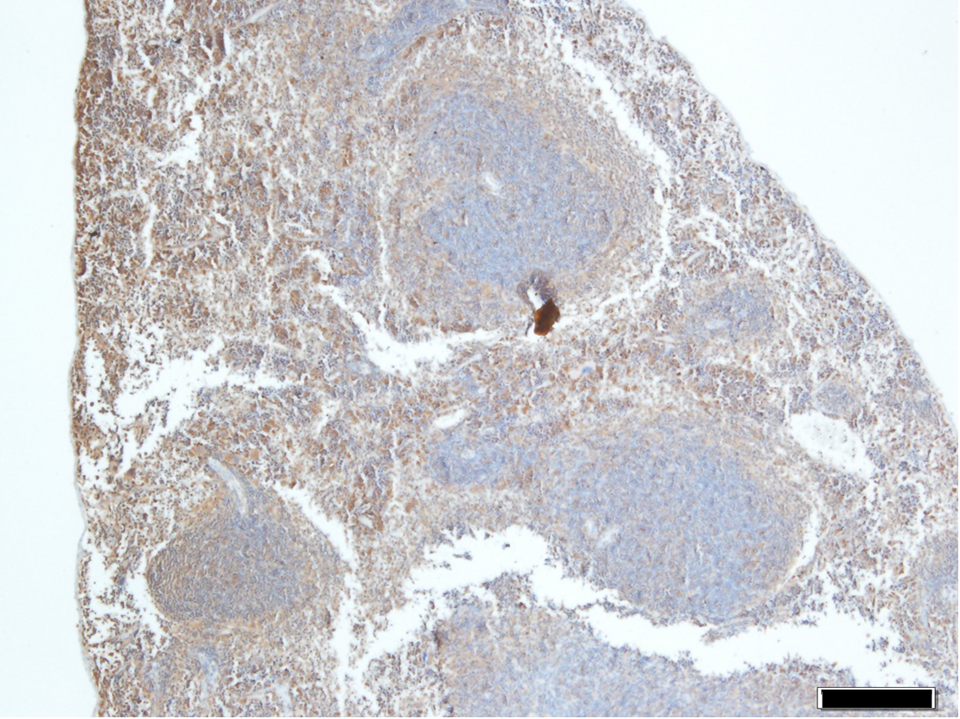

Supplement: S1 Fig — Bar, 200 μm. (TIF) [file pntd.0007816.s001.tif]

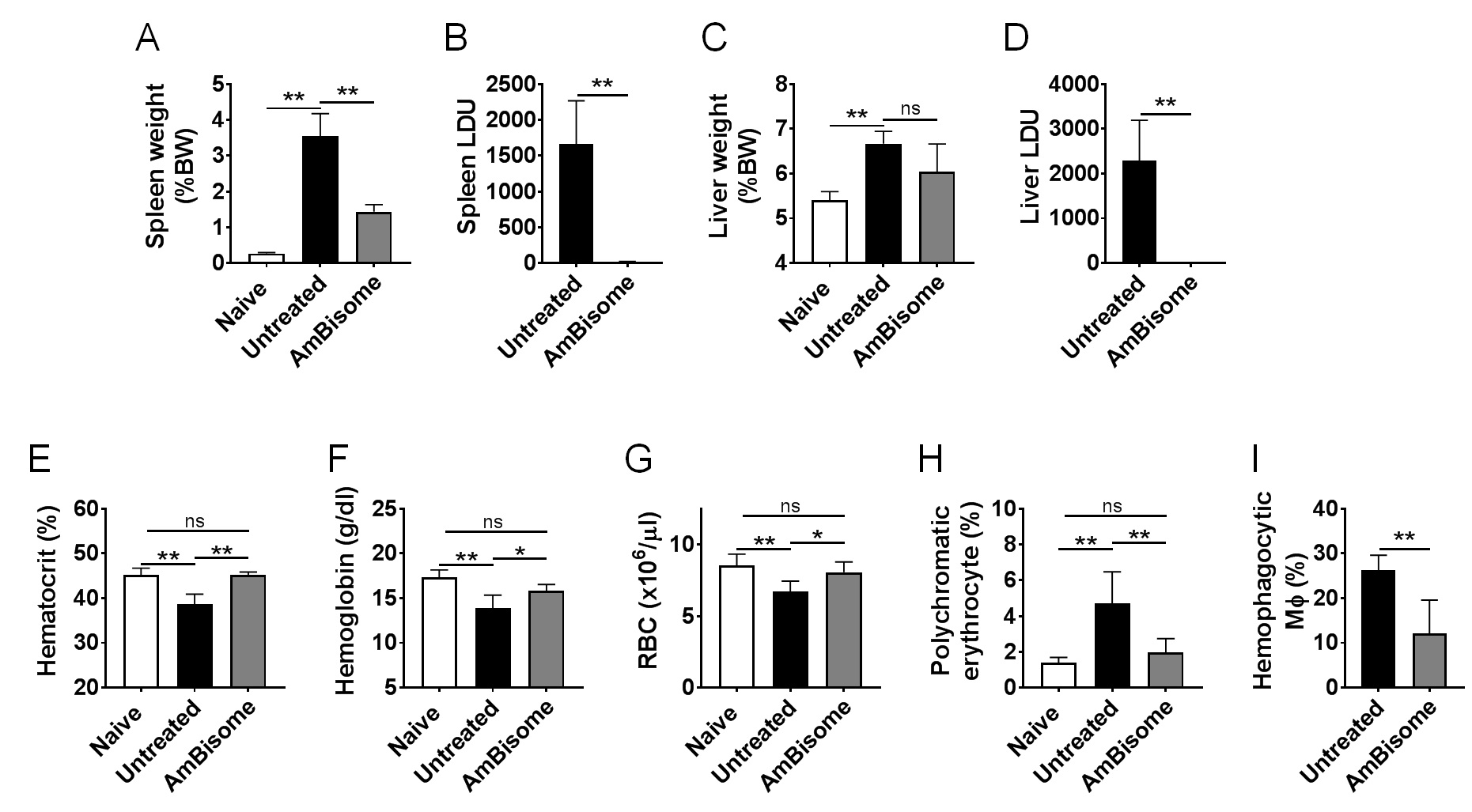

Supplement: S2 Fig — At 24 weeks post-infection, the mice were administered with 200 μg of liposomal amphotericin B (AmBisome; Dainippon Sumitomo Pharma, Japan) for 5 days. The mice were sacrificed 10 days after completing treatment to examine organ weights and parasite burden of the spleen (A and B) and the liver (C and D), in addition to hematocrit (E), hemoglobin (F), peripheral blood red blood cell counts (G), proportion of polychromatic erythrocytes in peripheral blood (H) and percentage of hemophagocytes out of total number of splenic macrophages (I). White, black and grey bars represent naïve, infected/untreated and infected/AmBisome-treated mice respectively. The mean and SD of 5 mice in each group are shown. This experiment was conducted once. *P < 0.05, **P < 0.01 by one-way ANOVA followed by Bonferroni's multiple comparisons test (for A, C, E to I) or unpaired t test (for B and D); ns, not significant. (TIF) [file pntd.0007816.s002.tif]

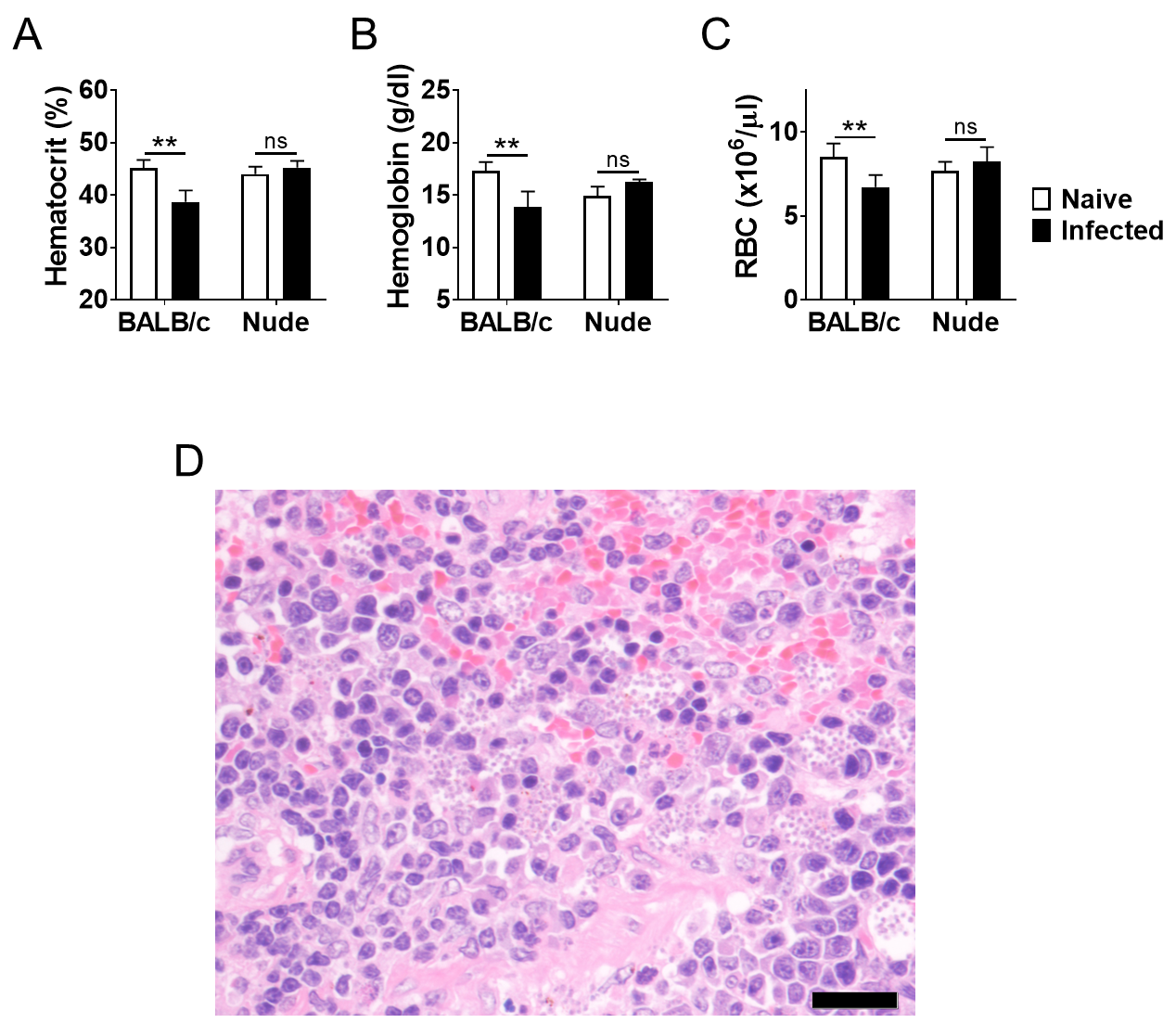

Supplement: S3 Fig — BALB/c mice and BALB/c-nu/nu (nude) mice (Clea) were infected with 1 × 10^7^ L. donovani promastigotes by intravenous injection into the tail vein. At 24 weeks post-infection, the infected mice as well as age-matched naïve mice were sacrificed to examine hematocrit (A), hemoglobin (B) and peripheral blood cell counts (C). The mean and SD of at least 4 mice in each group are shown. (D) A representative image of a HE-stained section of the spleen harvested from L. donovani-infected nude mice is shown. These are representative of two independent experiments with similar results. **P < 0.01 by two-way ANOVA followed by Bonferroni's multiple comparisons test; ns, not significant. (TIF) [file pntd.0007816.s003.tif]

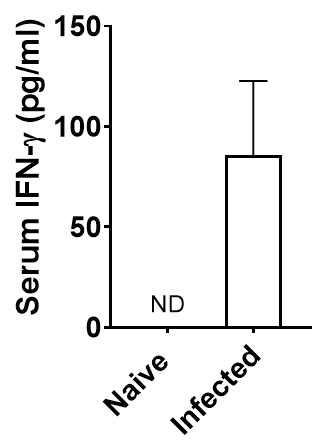

Supplement: S4 Fig — At 24 weeks post-infection, serum samples of naïve and infected mice were collected, and serum levels of IFN-γ were determined by using Mouse IFN gamma ELISA Ready-SET-Go! Kit (eBioscience, detection limit = 15 pg/ml). The mean and SD of 5 mice in each group are shown. ND, not detected. This experiment was conducted once. (TIF) [file pntd.0007816.s004.tif]
