# Supplementary material for: Hemophagocytosis induced by Leishmania donovani infection is beneficial to parasite survival within macrophages
Source: PLoS Negl Trop Dis. 2019 Nov 18;13(11):e0007816. doi: 10.1371/journal.pntd.0007816 (PMC6886864; doi:10.1371/journal.pntd.0007816)
Supplement: S1 Table — (DOCX) [file pntd.0007816.s005.docx]

**Table S1**. Primers used in this study

| Primer | Sequence | Gene | Reference |
| --- | --- | --- | --- |
| hmox1_F | CACGCATATACCCGCTACCT | mouse heme oxigenase 1 | Gobert, 2014,  J Immunol |
| hmox1_R | CCAGAGTGTTCATTCGAGCA |  |  |
| fpn1_F | CTACCATTAGAAGGATTGACCAGCT | mouse solute carrier family 40 (iron-regulated transporter), member 1 (Slc40a1) | Othman, 2014,  PLoS Pathog |
| fpn1_R | ACTGGAGAACCAAATGTCATAATCTG |  |  |
| fth1_F | GCGAGGTGGCCGAATCT | mouse ferritin heavy chane peptide 1 | Othman, 2014,  PLoS Pathog |
| fth1_R | CAGCCCGCTCTCCCAGT |  |  |
| Leish gapdh_F | CGCTGATCACGACCTTCTTC | *Leishmania donovani* glyceraldehyde 3-phosphate dehydrogenase, glycosomal | Bhandari, 2014, Antimicrob Agents Chemother |
| Leish gapdh_R | GAAGTACACGGTGGAGGCTG |  |  |
| gapdh_F | CGACTTCAACAGCAACTCCCACTCTTCC | mouse glyceraldehyde-3-phosphate dehydrogenase | Liu, 2009, Proc Natl Acad Sci USA |
| gapdh_R | TGGGTGGTCCAGGGTTTCTTACTCCTT |  |  |
