# Supplementary material for: Hemophagocytosis induced by Leishmania donovani infection is beneficial to parasite survival within macrophages
Source: PLoS Negl Trop Dis. 2019 Nov 18;13(11):e0007816. doi: 10.1371/journal.pntd.0007816 (PMC6886864; doi:10.1371/journal.pntd.0007816)
Supplement: S2 Table — (DOCX) [file pntd.0007816.s006.docx]

**Table S2**. mRNA levels of fHLH-involved genes in *L. donovani-*infected macrophages

| Gene name | Infected (A) | Naïve (B) | Log_2_ fold change (A/B) | Adjusted  *P* value |
| --- | --- | --- | --- | --- |
| Stx11 | 28.2 | 17.7 | 0.67 | 4.5.E-01 |
| Ap3b1 | 415.6 | 352.2 | 0.24 | 7.0.E-01 |
| Lyst | 616.7 | 491.4 | 0.33 | 9.0.E-01 |
| Xiap | 311.4 | 331.8 | -0.09 | 1.0.E+00 |
| Rab27a | 11.5 | 16.3 | -0.51 | 1.0.E+00 |
| Unc13d | 25.9 | 24.1 | 0.11 | 1.0.E+00 |
| Stxbp2 | 457.0 | 465.1 | -0.03 | 1.0.E+00 |
| Prf1 | 0.0 | 0.0 | 0.00 | 1.0.E+00 |
| Sh2d1a | 0.0 | 0.0 | 0.00 | 1.0.E+00 |
