# Supplementary material for: Hemophagocytosis induced by Leishmania donovani infection is beneficial to parasite survival within macrophages
Source: PLoS Negl Trop Dis. 2019 Nov 18;13(11):e0007816. doi: 10.1371/journal.pntd.0007816 (PMC6886864; doi:10.1371/journal.pntd.0007816)
Supplement: S3 Table — (DOCX) [file pntd.0007816.s007.docx]

**Table S3**. mRNA levels of phagocytosis-promoting receptor genes in *L. donovani*-infected macrophages

| Gene name | Infected (A) | Naïve (B) | log_2_ fold change (A/B) | Adjusted *P* value | Type |
| --- | --- | --- | --- | --- | --- |
| Fcgr1 | 378.8 | 86.7 | 2.13 | 1.3.E-04 | FcR |
| Tlr7 | 314.2 | 719.0 | -1.19 | 7.0.E-04 | TLR |
| Itgav | 334.3 | 165.5 | 1.01 | 8.8.E-04 | vitronectin R |
| Cd36 | 2338.2 | 1142.9 | 1.03 | 2.3.E-03 | Scavenger R |
| Tlr2 | 672.6 | 1491.3 | -1.15 | 2.4.E-03 | TLR |
| Cxcl16 | 179.1 | 513.3 | -1.52 | 3.0.E-03 | Scavenger R |
| Fcgr4 | 126.2 | 62.6 | 1.01 | 6.1.E-03 | FcR |
| Scarb2 | 408.5 | 249.8 | 0.71 | 1.1.E-02 | Scavenger R |
| Cd14 | 1681.8 | 2596.0 | -0.63 | 2.9.E-02 | TLR |
| Scarb1 | 171.1 | 267.0 | -0.64 | 3.7.E-02 | Scavenger R |
| Msr1 | 1184.1 | 1655.7 | -0.48 | 1.2.E-01 | Scavenger R |
| Tlr9 | 17.1 | 55.9 | -1.71 | 1.3.E-01 | TLR |
| Tlr13 | 437.0 | 607.2 | -0.47 | 1.6.E-01 | TLR |
| Sra1 | 205.1 | 164.4 | 0.32 | 5.7.E-01 | Scavenger R |
| Tlr3 | 35.3 | 22.5 | 0.65 | 6.1.E-01 | TLR |
| Tlr1 | 49.6 | 58.9 | -0.25 | 8.0.E-01 | TLR |
| Itgam | 2.4 | 5.2 | -1.14 | 8.0.E-01 | CR |
| Scarf1 | 23.6 | 32.7 | -0.47 | 8.2.E-01 | Scavenger R |
| Ssc5d | 4.0 | 7.3 | -0.88 | 8.7.E-01 | Scavenger R |
| Fcgr3 | 479.2 | 420.2 | 0.19 | 9.4.E-01 | FcR |
| Clec7a | 126.9 | 144.1 | -0.18 | 9.6.E-01 | c-lectin R |
| Tlr6 | 45.3 | 51.7 | -0.19 | 1.0.E+00 | TLR |
| Ssc4d | 5.7 | 4.6 | 0.30 | 1.0.E+00 | Scavenger R |
| Olr1 | 1.6 | 0.9 | 0.76 | 1.0.E+00 | Scavenger R |
| Mrc1 | 1.0 | 1.7 | -0.84 | 1.0.E+00 | c-lectin R |
| Tlr4 | 395.2 | 391.7 | 0.01 | 1.0.E+00 | TLR |
| Tlr8 | 16.3 | 12.8 | 0.34 | 1.0.E+00 | TLR |
| Itgb2 | 2200.9 | 2231.1 | -0.02 | 1.0.E+00 | CR |
| Scarf2 | 0.0 | 0.4 | -10.00 | 1.0.E+00 | Scavenger R |
| Tlr5 | 0.2 | 0.2 | 0.11 | 1.0.E+00 | TLR |
| Cr2 | 0.0 | 0.0 | 0.00 | 1.0.E+00 | CR |
| Tlr11 | 0.0 | 0.0 | 0.00 | 1.0.E+00 | TLR |
| Tlr12 | 0.0 | 0.0 | 0.00 | 1.0.E+00 | TLR |
| Cd209a | 0.0 | 0.0 | 0.00 | 1.0.E+00 | c-lectin R |
| Marco | 0.0 | 0.0 | 0.00 | 1.0.E+00 | Scavenger R |
| Fcrls | 0.0 | 0.0 | 0.00 | 1.0.E+00 | Scavenger R |
| Scara3 | 0.0 | 0.0 | 0.00 | 1.0.E+00 | Scavenger R |
| Scara5 | 0.0 | 0.0 | 0.00 | 1.0.E+00 | Scavenger R |
