# Supplementary material for: Hemophagocytosis induced by Leishmania donovani infection is beneficial to parasite survival within macrophages
Source: PLoS Negl Trop Dis. 2019 Nov 18;13(11):e0007816. doi: 10.1371/journal.pntd.0007816 (PMC6886864; doi:10.1371/journal.pntd.0007816)
Supplement: S4 Table — (DOCX) [file pntd.0007816.s008.docx]

**Table S4**. mRNA levels of phagocytosis-inhibitory receptor genes in *L. donovani*-infected macrophages

| Gene name | Infected (A) | Naïve (B) | log_2_ fold change (A/B) | Adjusted  *P* value | Ligand |
| --- | --- | --- | --- | --- | --- |
| Cd33 | 313.0 | 778.1 | -1.31 | 5.7.E-10 | sialic acid |
| Cd200r4 | 130.2 | 17.4 | 2.91 | 2.4E-06 | CD200 |
| Adgre5 | 71.1 | 145.4 | -1.03 | 8.2E-02 | CD55 |
| Fcgr2b | 288.5 | 535.3 | -0.89 | 9.3.E-02 | IgG |
| Cd200r1 | 161.2 | 75.9 | 1.09 | 1.3E-01 | CD200 |
| Siglece | 5.4 | 17.3 | -1.67 | 1.7.E-01 | sialic acid |
| Pirb | 353.8 | 585.0 | -0.73 | 2.2.E-01 | ? |
| Cd200r2 | 15.2 | 5.6 | 1.44 | 4.6E-01 | CD200 |
| Sirpa | 3840.9 | 4310.7 | -0.17 | 9.5E-01 | CD47 |
| Pilra | 5.8 | 7.7 | -0.42 | 1.0.E+00 | ? |
| Cd200r3 | 3.3 | 2.2 | 0.56 | 1.0.E+00 | CD200 |
| Siglec1 | 168.1 | 182.5 | -0.12 | 1.0.E+00 | sialic acid |
| siglec | 1.5 | 2.1 | -0.47 | 1.0.E+00 | sialic acid |
| Pecam1 | 1.1 | 0.5 | 1.20 | 1.0.E+00 | ? |
| Lilr4b | 0.6 | 0.4 | 0.65 | 1.0.E+00 | ? |
| Klrg1 | 0.0 | 0.0 | 0.00 | 1.0.E+00 | ? |
